# In Vitro Evaluation of the Antimicrobial Activity of Chlorhexidine-Based Shampoos Against Clinical Isolates of *Malassezia pachydermatis* and Multidrug-Resistant *Staphylococcus pseudintermedius*

**DOI:** 10.3390/microorganisms14081660

**Published:** 2026-07-29

**Authors:** Marcela de Oliveira Platenik, Gianna Goldman, Domenico Santoro

**Affiliations:** 1 Department of Small Animal Clinical Sciences, University of Florida College of Veterinary Medicine, Gainesville, FL 32610, USA; marceladeoliveir@ufl.edu; 2 Department of Pathobiology, University of Pennsylvania School of Veterinary Medicine, Philadelphia, PA 19104, USA; goldmang@vet.upenn.edu

**Keywords:** *Staphylococcus pseudintermedius*, *Malassezia pachydermatis*, chlorhexidine, antimicrobial susceptibility, topicals

## Abstract

*Staphylococcus pseudintermedius* (*SP*), including multidrug-resistant *SP* (MDR-*SP*), and *Malassezia pachydermatis* (*MP*) are major canine cutaneous pathogens. Chlorhexidine-based shampoos, alone or containing azoles, are commonly used in veterinary dermatology; however, comparative data on commercially available products are limited. This study evaluated the in vitro antimicrobial activity of seven chlorhexidine-based shampoos against 30 clinical MDR-*SP* isolates and 30 clinical *MP* isolates. Six two-fold dilutions (1:16,000–1:512,000) of KetoChlor^®^, BioHex^®^, MAX Chlorhexidine 4%, Douxo S3™ Pyo, TrizCHLOR^®^4, Malaseb^®^, and MiconaHex+Triz^®^ were tested using a broth microdilution assay. Minimum inhibitory, bactericidal, and fungicidal concentrations (MIC, MBC, and MFC) were determined. Malaseb^®^ and MiconaHex+Triz^®^ exhibited the lowest MIC and MBC/MFC values, although the observed differences represented a single two-fold dilution step, with MIC values of 1:128,000 against both MDR-*SP* and *MP*, and MBC/MFC values of 1:32,000 and 1:128,000, respectively. The remaining shampoos demonstrated bactericidal activity (MIC = 1:32,000/1:64,000; MBC = 1:16,000), while some exhibited MIC and MFC values of 1:64,000 against *MP*. MIC and MFC endpoints were not reached for a limited number of *MP* isolates. Overall, all shampoo formulations demonstrated substantial antimicrobial activity across the tested dilution range. Further clinical studies are needed to determine whether these in vitro differences translate into meaningful in vivo efficacy.

## 1. Introduction

Skin infections are one of the most common reasons for antimicrobial prescriptions in both human and veterinary practices. *Staphylococcus pseudintermedius* (*SP*) and *Malassezia pachydermatis* (*MP*) are common opportunistic causes of skin infections in dogs. *Staphylococcus pseudintermedius* is the most common cause of canine pyoderma, while *MP* is the most common yeast isolated from the ears and skin in canine patients [1,2]. Multidrug resistance (MDR) [3], specifically in *SP*, has become an important concern in canine dermatology. Similarly, *MP* isolates with reduced susceptibility or antifungal resistance, particularly to azole antifungals, have also been increasingly reported [2]. It has been hypothesized that the increase in antimicrobial resistance in both organisms has been exacerbated by the indiscriminate use of systemic antimicrobials. One of the factors driving resistance is the overuse and/or misuse of systemic antimicrobials [4,5]. Due to increasing rates of antibiotic and antifungal resistance, topical treatments are recommended as the first choice for surface and superficial infections [6].

The umbrella of “topical treatments” includes the use of shampoos, mousse, sprays, and wipes, often containing chlorhexidine or other antiseptics. Chlorhexidine-based shampoos contain varying chlorhexidine salts (gluconate/digluconate, acetate, and hydrochloride salts) and concentrations ranging from 2% to 4% [7]. Chlorhexidine digluconate, the most used formulation, is generally recognized as an effective treatment for both Gram-positive and Gram-negative organisms as well as *MP*. Its main mechanism of action is the disruption of the organisms’ cell membrane, resulting in leakage of intracellular components [1]. Commercially available chlorhexidine-based products usually combine chlorhexidine with an azole; however, products containing only chlorhexidine are also available. Similar antimicrobial efficacy has been clinically observed among products.

Previous in vitro studies demonstrated that the presence of azoles increases the efficacy of chlorhexidine [1,7]. Similarly, previous in vitro studies have shown that different chlorhexidine-based shampoos may act similarly independent of the concentration of chlorhexidine or the additional presence of antifungals [7]. However, data are limited to the simultaneous comparison of those products against MDR-*SP* and/or *MP* clinical isolates. Shampoos offer a cost-effective and safe method of treating pyoderma while hydrating the skin, providing major help in the management of allergic disease [8].

The objective of this study was to compare in vitro antimicrobial activity of seven commercially available chlorhexidine-based shampoos independently against clinical isolates of MDR-*SP* and *MP*. The selected shampoos include KetoChlor^®^ (Virbac; Fort Worth, TX, USA), BioHex^®^ (Nextmune; Phoenix, AZ, USA), MAX Chlorexidine 4% (Anmpharm; North Augusta, SC, USA), Douxo S3^TM^ Pyo (Ceva Animal Health; Lenexa, KS, USA), TrizCHLOR^®^4 (Dechra; Overland Park, KS, USA), Malaseb^®^ (Dechra), and MiconaHex+Triz^®^ (Dechra). Although *SP* and *MP* differ in their biology, growth requirements, susceptibility testing conditions, and mechanisms of antimicrobial resistance, they are the most common pathogens associated with canine superficial skin infections, coexisting in cases of pyoderma and otitis externa. For this reason, a secondary aim of the study was to compare how these shampoos perform against MDR-*SP* and *MP*.

## 2. Materials and Methods

### 2.1. Microbial Isolation and Identification

A total of sixty clinical isolates were included in this study, including 30 MDR-*SP* isolates recovered from canine skin lesions and 30 *MP* clinical isolates collected from canine skin and ear samples. Each isolate represented a single clinical isolate obtained from a different dog presented to the University of Florida Veterinary Hospital. Duplicate isolates from the same patient or the same episode were not included. The antimicrobial resistance profiles of the MDR-*SP* had been previously determined according to published guidelines [3,9,10], and are summarized in Supplementary Table S1. The isolates were obtained from the Clinical Microbiology Service at the authors’ institution and stored in glycerol beads. The identification of each isolate was performed based on biochemical features identified using colony morphology with the Trek Sensitre (Trek Diagnostic Systems, Inc.; Cleveland, OH, USA) automated system, according to Clinical and Laboratory Standards Institute (CLSI) guidelines (Clinical and Laboratory Standards Institute, 4th Edition), and confirmed using matrix-assisted laser desorption/ionization time-of-flight (MALDI-TOF) mass spectrometry.

### 2.2. Isolates Preparation

Each assay had one American Type Culture Collection (ATCC) strain as an internal control: ATCC 49444 *SP* and ATCC 14522 *MP*. The bacterial samples were subcultured on Columbia Agar Plates with 5% sheep blood (Hardy Diagnostics; Santa Maria, CA, USA) at 37 °C for 24 h. The yeast samples were subcultured on Sabouraud dextrose agar (Hardy Diagnostics) at 37 °C for 72 h. Colonies were then inoculated into sterile water at 0.5 Mc Farland [~1 × 10^8^ colony forming units (CFUs)/mL for *SP* and ~1 × 10^6^ CFU/mL for *MP*] Standard using the Den-1B Mc Farland Densitometer (Grant Instruments; Shepreth, Cambridgeshire, UK). The inoculum was diluted into Sensitre^TM^ cation-adjusted Mueller-Hinton Broth (MHB) with TES broth (Remel, San Diego, CA, USA) to reach a concentration of 1 × 10^6^ CFUs/mL for *SP* and 1 × 10^4^ CFUs/mL for *MP.* The test medium was selected based on previously published protocols and prior validation in our laboratory, which demonstrated adequate and reproducible growth of MP under these conditions [1,11].

### 2.3. Treatment Preparation

Shampoo formulations include KetoChlor^®^ (2% chlorhexidine gluconate + 1% ketoconazole, Virbac), BioHex^®^ (2% chlorhexidine gluconate + 2% miconazole + microsilver, Nextmune), MAX Chlorexidine 4% (4% chlorhexidine gluconate, Anmpharm), Douxo 3S^TM^ Pyo (3% chlorhexidine digluconate + 0.5% ophytrium, Ceva Animal Health), TrizCHLOR^®^4 (4% chlorhexidine gluconate, Dechra), Malaseb^®^ (2% chlorhexidine gluconate + 2% miconazole, Dechra), and MiconaHex+Triz^®^ (2% chlorhexidine gluconate + 2% miconazole + tris EDTA, Dechra).

### 2.4. Microbroth Dilution Assay

A microbroth dilution (MBD) methodology was performed to determine the minimum inhibitory concentration (MIC). This assay is used to determine the lowest concentration of an antimicrobial agent (shampoo) required to inhibit the growth of the organism (MDR-*SP* and *MP*). The MIC_50_ and MIC_90_ indicating the minimum concentration able to inhibit 50% and 90% of the bacterial/fungal isolates were also calculated. Based on previous studies [11] and initial trials using ATCC isolates (ATCC 49444 and ATCC 14522), six two-fold dilutions (final concentrations tested ranged from 1:16,000 to 1:512,000) for each shampoo were tested. The ATCC strains were also used as internal experimental controls.

To determine the MIC, the MBD was performed using 96-well microtiter plates with round bottom wells, following the CLSI guidelines [9]. Briefly, 100 µL of each shampoo dilution was delivered to each well. Then, 100 µL of the bacterial/fungal inoculum was added to each well, reaching a final inoculum concentration of 5 × 10^5^ CFUs/mL per well for MDR-*SP* and 5 × 10^3^ CFUs/mL per well for *MP*. Plates were incubated for 24 h (MDR-*SP*) or 72 h (*MP*) at 37 °C according to previously published susceptibility testing methods and preliminary studies not reported here [1,10].

A total of 100 µL of broth and 100 µL of the inoculum were delivered to the positive control wells (growth control). Negative controls consisted of wells containing 100 µL of sterile water and 100 µL of broth (contamination control). Additional control wells containing each shampoo and broth without microbial inoculum were tested to verify the sterility of the shampoo preparations and to identify any background turbidity or visual interference during endpoint determination. At the dilutions tested, none of the shampoo formulations exhibited visible interference with MIC determination.

The MIC was reported as having the lowest concentration without visible growth. All samples were run in duplicates as technical replicates using the same inoculum preparation. The discrepancies between duplicate wells were allowed, and in this case, the higher concentration was considered. The median value of each clinical isolate was used for statistical analysis.

### 2.5. Minimum Bactericidal (or Fungicidal) Concentration Assay

This assay was used to determine the lowest concentration of the antimicrobial agent (shampoo) able to kill the organism (MDR-*SP* and *MP*). The MBC/MFC_50_ and MBC/MFC_90_ indicating the minimum concentration able to kill 50% and 90% of the bacterial/fungal isolates were also calculated. Measurement of the MBC/MFC was performed by inoculating 100 µL of the MIC and at least two dilutions above the MIC on Columbia Agar Plates with 5% sheep blood or Sabouraud dextrose agar, which were incubated for 24 h (MDR-*SP*) and 72 h (*MP*) at 37 °C. The MBC/MFC was reported as the highest dilution that did not allow colony growth.

### 2.6. Ethics Statement

This study does not involve animals. The microorganisms were banked via the clinical microbiology laboratory.

### 2.7. Statistical Analysis

Data were analyzed to demonstrate the behavior of different shampoos using the same isolates of MDR-*SP* and *MP*. Data were tested for normality using the Shapiro–Wilk test (α = 0.05). The Friedman test followed by Dunn’s multiple comparison test was used to compare and assess the efficacy of each shampoo. MIC_50_, MIC_90_, MBC_50_, MBC_90_, MFC_50_ and MFC_90_ values were calculated using the observed values from all isolates with an achievable MIC, MBC, or MFC. Isolates that did not reach an MIC, MBC, or MFC endpoint within the tested dilution range were treated as censored observations and were not considered for statistical analysis. The final number of isolates statistically analyzed for MDR-SP (MIC: *n* = 30; MBC: *n* = 27) and MP (MIC: *n* = 14; MFC: *n* = 14) varied. The Mann–Whitney U test was also used to assess the efficacy of each shampoo against MDR-*SP* and *MP*. Significance was set at *p* < 0.05. Comparisons were performed using GraphPad Prism 10.2.3 software (GraphPad Software Inc.; La Jolla, CA, USA).

## 3. Results

### 3.1. MDR-SP Isolates (Table 1; Figure 1)

The median MIC, MBC, as well as the MIC_50/90_ and MBC_50/90_ for each shampoo are listed in Table 1. The median MIC was 1:128,000 for Malaseb^®^ and MiconaHex+Triz^®^, 1:64,000 for BioHex^®^, MAX Chlorexidine 4%, and TrizCHLOR^®^4, and 1:32,000 for KetoChlor^®^ and Douxo S3^TM^ Pyo. Statistically, there was no difference between Malaseb^®^ and MiconaHex+Triz^®^ (*p* > 0.999). However, Malaseb^®^ (Malaseb^®^ vs. TrizCHLOR^®^4: *p* = 0.044; Malaseb^®^ vs. BioHex^®^: *p* = 0.029; Malaseb^®^ vs. Douxo S3^TM^ Pyo: *p* < 0.0001; Malaseb^®^ vs. KetoChlor^®^: *p* < 0.0001; Malaseb^®^ vs. MAX Chlorexidine 4%: *p* = 0.0019) and MiconaHex+Triz^®^ (MiconaHex+Triz^®^ vs. TrizCHLOR^®^4: *p* = 0.039; MiconaHex+Triz^®^ vs. BioHex^®^: *p* = 0.026; MiconaHex+Triz^®^ vs. Douxo S3^TM^ Pyo: *p* < 0.0001; MiconaHex+Triz^®^ vs. KetoChlor^®^: *p* < 0.0001; MiconaHex+Triz^®^ vs. MAX Chlorexidine 4%: *p* = 0.0017) had a significantly lower MIC than the other shampoos. In addition, MAX Chlorhexidine 4%, BioHex^®^, and Douxo S3^TM^ Pyo had a lower MIC than KetoChlor^®^ (*p* = 0.0003; *p* < 0.0001; *p* = 0.044, respectively).

**Table 1:** Antimicrobial susceptibility results expressed as median minimum inhibitory concentration (MIC), median minimum bactericidal concentration (MBC), and their respective 50% (MIC/MBC_50_) and 90% (MIC/MBC_90_) values of MDR-SP isolates. The corresponding chlorhexidine concentration is reported in brackets.

Shampoo	Median MIC	MIC_50_	MIC_90_	Median MBC	MBC_50_	MBC_90_
MAX Chlorhexidine 4^®^	1:64,000 (0.625 µg/mL)	1:64,000 (0.625 µg/mL)	1:64,000 (0.625 µg/mL)	1:16,000 (2.5 µg/mL)	1:16,000 (2.5 µg/mL)	1:16,000 (2.5 µg/mL)
KetoChlor^®^	1:32,000 (0.625 µg/mL)	1:32,000 (0.625 µg/mL)	1:16,000 (1.25 µg/mL)	1:16,000 (1.25 µg/mL)	1:16,000 (1.25 µg/mL)	1;16,000 (1.25 µg/mL)
Malaseb^®^	1:128,000 (0.156 µg/mL)	1:128,000 (0.156 µg/mL)	1:128,000 (0.156 µg/mL)	1:32,000 (0.625 µg/mL)	1:32,000 (0.625 µg/mL)	1:32,000 (0.625 µg/mL)
MiconaHex+Triz^®^	1:128,000 (0.156 µg/mL)	1:128,000 (0.156 µg/mL)	1:128,000 (0.156 µg/mL)	1:32,000 (0.625 µg/mL)	1:32,000 (0.625 µg/mL)	1:32,000 (0.625 µg/mL)
TrizCHLOR^®^4	1:64,000 (0.625 µg/mL)	1:64,000 (0.625 µg/mL)	1:64,000 (0.625 µg/mL)	1:16,000 (2.5 µg/mL)	1:16,000 (2.5 µg/mL)	1:16,000 (2.5 µg/mL)
BioHex^®^	1:64,000 (0.3125 µg/mL)	1:64,000 (0.3125 µg/mL)	1:64,000 (0.3125 µg/mL)	1:16,000 (1.25 µg/mL)	1:16,000 (1.25 µg/mL)	1:16,000 (1.25 µg/mL)
Douxo S3^TM^ Pyo	1:32,000 (0.937 µg/mL)	1:32,000 (0.937 µg/mL)	1:32,000 (0.937 µg/mL)	1:16,000 (1.87 µg/mL)	1:16,000 (1.87 µg/mL)	1:16,000 (1.87 µg/mL)

The MIC_50_ and MIC_90_ of Malaseb^®^ and MiconaHex+Triz^®^ were set at 1:128,000. The MIC_50_ of all the other shampoos was one or two dilutions lower, ranging between 1:64,000 (MAX Chlorexidine 4%, TrizCHLOR^®^4, and BioHex^®^) and 1:32,000 (KetoChlor^®^ and Douxo S3^TM^ Pyo). The MIC_90_ of all shampoos was the same as their MIC_50_ with the exception of KetoChlor^®^, for which the MIC_90_ was one dilution lower than the MIC_50_ (MIC_90_ of 1:16,000).

With regard to the MBC, five shampoos (KetoChlor^®^, BioHex^®^, MAX Chlorexidine 4%, Douxo S3^TM^ Pyo, and TrizCHLOR^®^4) had their MBC, MBC_50_, and MBC_90_ set at 1:16,000. The median MBC, MBC_50_, and MBC_90_ for Malaseb^®^ and MiconaHex+Triz^®^ was one dilution above (1:32,000) the other shampoos. Similarly to the MIC, there was no difference between the MBC of Malaseb^®^ and MiconaHex+Triz^®^ (*p* > 0.999). However, Malaseb^®^ (Malaseb^®^ vs. TrizCHLOR^®^4: *p* = 0.001; Malaseb^®^ vs. BioHex^®^: *p* = 0.0008; Malaseb^®^ vs. Douxo S3^TM^ Pyo: *p* = 0.0004; Malaseb^®^ vs. KetoChlor^®^: *p* < 0.0001; Malaseb^®^ vs. MAX Chlorexidine 4%: *p* < 0.0001) and MiconaHex+Triz^®^ (MiconaHex+Triz^®^ vs. TrizCHLOR^®^4: *p* = 0.0007; MiconaHex+Triz^®^ vs. BioHex^®^: *p* = 0.0037; MiconaHex+Triz^®^ vs. Douxo S3^TM^ Pyo: *p* = 0.002; MiconaHex+Triz^®^ vs. KetoChlor^®^: *p* < 0.0001; MiconaHex+Triz^®^ vs. MAX Chlorexidine 4%: *p* < 0.0001) had a significantly lower MBC than the other shampoos. There was no difference in MBC values among the other five shampoos (*p* > 0.62). The MBC_50_ and MBC_90_ of Malaseb^®^ and MiconaHex+Triz^®^ were set at 1:32,000. The MBC_50_ and MBC_90_ of all the other shampoos were one dilution lower at 1:16,000 (MAX Chlorexidine 4%, TrizCHLOR^®^4, and BioHex^®^, KetoChlor^®^, and Douxo S3^TM^ Pyo). An MBC was not achievable for two isolates exposed to KetoChlor^®^ and one isolate exposed to BioHex^®^.

The *MDR-SP* ATCC strain (*SP*-49444), inclued as an internal quality control, generated similar results. Indeed, Malaseb^®^, MiconaHex+Triz^®^, and BioHex^®^ demonstrated MIC values of 1:128,000, whereas Douxo S3^TM^ Pyo, TrizCHLOR^®^4, and MAX Chlorexidine 4% demonstrated MIC values of 1:64,000. KetoChlor^®^ exhibited a MIC of 1:32,000. MBC values were 1:64,000 for Malaseb^®^ and MiconaHex+Triz^®^. MAX Chlorexidine 4%, TrizCHLOR^®^4, and BioHex^®^ had MBC values of 1:32,000. KetoChlor^®^ and Douxo S3^TM^ Pyo had MBC values of 1:16,000.

**Figure 1:**
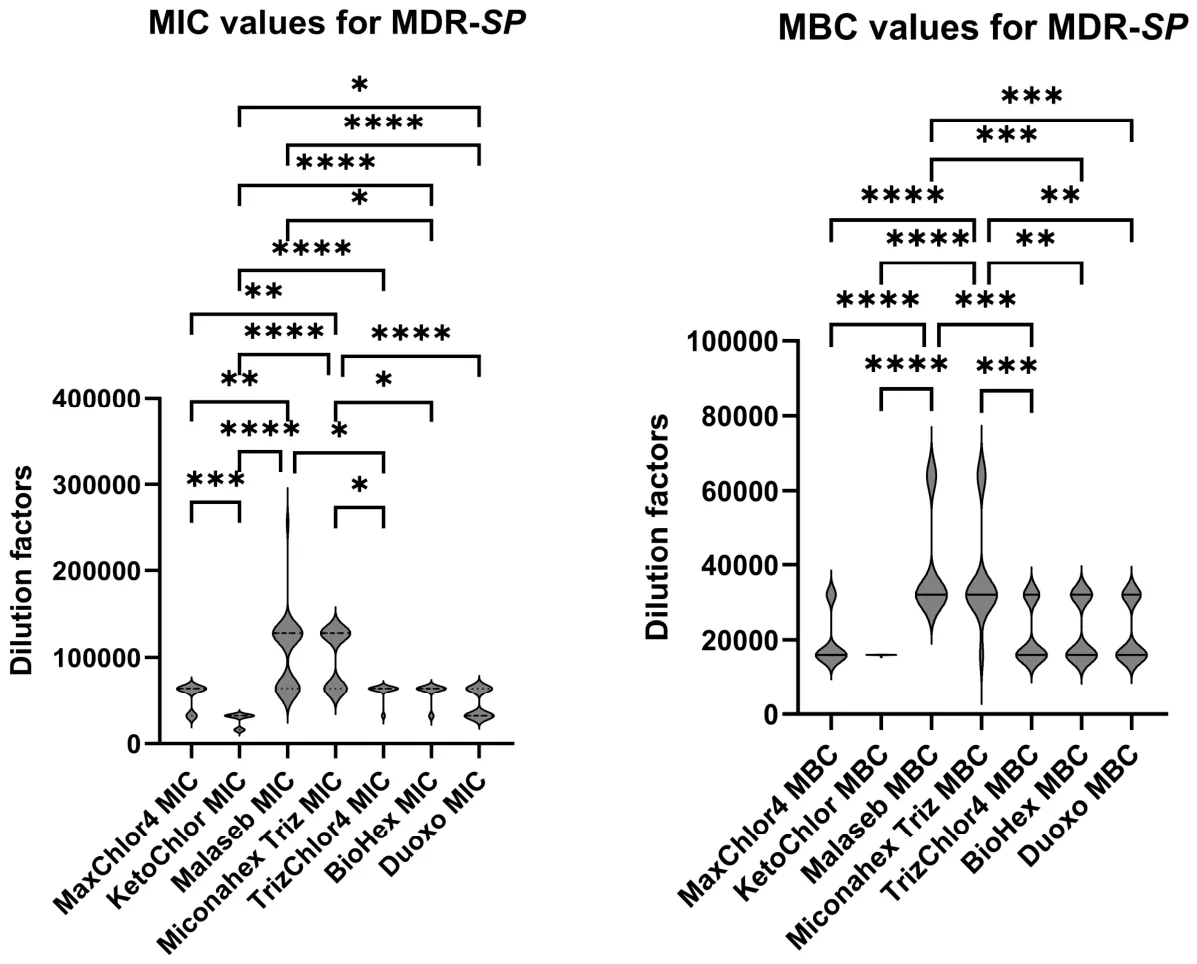
Violin plot representing comparisons of the minimum inhibitory concentration (MIC) and minimum bactericidal concentration (MBC) of MDR-SP isolates. * *p* < 0.05; ** *p* < 0.01; *** *p* < 0.001; **** *p* < 0.0001. MDR-SP: multidrug-resistant *Staphylococcus pseudintermedius*.

### 3.2. MP Isolates (Table 2; Figure 2)

The median MIC, MFC, MIC_50/90_, and MFC_50/90_ for each shampoo are listed in Table 2. The median MIC was 1:128,000 for Malaseb^®^ and MiconaHex+Triz^®^, whereas the remaining shampoos (KetoChlor^®^, BioHex^®^, MAX Chlorexidine 4%, TrizCHLOR^®^4, and Douxo S3^TM^ Pyo) had median MIC values of 1:64,000. However, when the MIC values of each shampoo were statistically compared, the only differences were noted between MiconaHex+Triz^®^ and MAX Chlorexidine 4% (*p* = 0.04), MiconaHex+Triz^®^ and Douxo S3^TM^ Pyo (*p* = 0.0059), and MiconaHex+Triz^®^ and BioHex^®^ (*p* = 0.046).

**Table 2:** Antifungal susceptibility results expressed as median minimum inhibitory concentration (MIC), median minimum fungicidal concentration (MFC), and their respective 50% (MIC/MFC_50_) and 90% (MIC/MFC_90_,) values of *MP* isolates. The corresponding chlorhexidine concentration is reported in brackets. MIC_90_ and MFC_90_ were reported only when at least 90% of the isolates reached the corresponding endpoint within the tested dilution range. Otherwise, these values were reported as not determined (ND).

Shampoo	Median MIC	MIC_50_	MIC_90_	Median MFC	MFC_50_	MFC_90_
MAX Chlorhexidine 4^®^	1:64,000 (0.625 µg/mL)	1:64,000 (0.625 µg/mL)	1:16,000 (2.5 µg/mL)	1:64,000 (0.625 µg/mL)	1:64,000 (0.625 µg/mL)	1:16,000 (2.5 µg/mL)
KetoChlor^®^	1:64,000 (0.3125 µg/mL)	1:64,000 (0.3125 µg/mL)	ND	1:64,000 (0.3125 µg/mL)	1:64,000 (0.3125 µg/mL)	ND
Malaseb^®^	1:128,000 (0.156 µg/mL)	1:128,000 (0.156 µg/mL)	1:32,000 (0.625 µg/mL)	1:128,000 (0.156 µg/mL)	1:128,000 (0.156 µg/mL)	1:32,000 (0.625 µg/mL)
MiconaHex+Triz^®^	1:128,000 (0.156 µg/mL)	1:128,000 (0.156 µg/mL)	1:32,000 (0.625 µg/mL)	1:128,000 (0.156 µg/mL)	1:128,000 (0.156 µg/mL)	1:32,000 (0.625 µg/mL)
Triz CHLOR 4^®^	1:64,000 (0.625 µg/mL)	1:64,000 (0.625 µg/mL)	ND	1:64,000 (0.625 µg/mL)	1:64,000 (0.625 µg/mL)	ND
BioHex^®^	1:64,000 (0.3125 µg/mL)	1:64,000 (0.3125 µg/mL)	ND	1:64,000 (0.3125 µg/mL)	1:64,000 (0.3125 µg/mL)	ND
Douxo S3^TM^ Pyo	1:64,000 (0.468 µg/mL)	1:64,000 (0.468 µg/mL)	1:32,000 (0.937 µg/mL)	1:64,000 (0.468 µg/mL)	1:64,000 (0.468 µg/mL)	1:32,000 (0.937 µg/mL)

The MIC_50_ for five shampoos (KetoChlor^®^, BioHex^®^, MAX Chlorexidine 4%, Douxo S3^TM^ Pyo, and TrizCHLOR^®^4) was also 1:64,000, whereas Malaseb^®^ and MiconaHex+Triz^®^ had an MIC_50_ of 1:128,000. Based on the analysis using only observed endpoints, MIC_90_ values were determined for Malaseb^®^ (1:32,000), MiconaHex+Triz^®^ (1:32,000), Douxo S3^TM^ Pyo (1:32,000), and MAX Chlorexidine 4% (1:16,000). The MIC_90_ could not be determined within the tested dilution range for TrizCHLOR^®^4, BioHex^®^, and KetoChlor^®^.

As for the MFC, all the shampoos performed similarly. Five shampoos (KetoChlor^®^, BioHex^®^, MAX Chlorexidine 4%, Douxo S3^TM^ Pyo, and TrizCHLOR^®^4) had the same MFC of 1:64,000, whereas Malaseb^®^ and MiconaHex+Triz^®^ had an MFC of 1:128,000. However, when the MFC values of each shampoo were statistically compared, there was no difference among them (*p* > 0.2).

The MFC_50_ was 1:128,000 for Malaseb^®^ and MiconaHex+Triz^®^, whereas all the remaining shampoos (KetoChlor^®^, BioHex^®^, MAX Chlorexidine 4%, Douxo S3^TM^ Pyo, and TrizCHLOR^®^4) had an MFC_50_ of 1:64,000. Based on the analysis using only observed endpoints, MFC_90_ values of 1:32,000 were determined for Malaseb^®^, MiconaHex+Triz^®^, and Douxo S3^TM^ Pyo and 1:16,000 for MAX Chlorexidine 4%. The MFC_90_ could not be determined within the tested dilution range for TrizCHLOR^®^4, BioHex^®^, and KetoChlor^®^.

Despite the high antifungal activity observed across all shampoos, a small number of *MP* isolates were not inhibited/killed at the dilutions tested. Specifically, an MIC and an MFC were not achieved for six isolates exposed to TrizCHLOR^®^4, five isolates exposed to BioHex^®^, four isolates exposed to KetoChlor^®^, two isolates exposed to Douxo S3^TM^ Pyo, two isolates exposed to Malaseb^®^, and one isolate exposed to MAX Chlorexidine 4%. All isolates exposed to MiconaHex+Triz^®^ reached both MIC and MFC endpoints.

**Figure 2:**
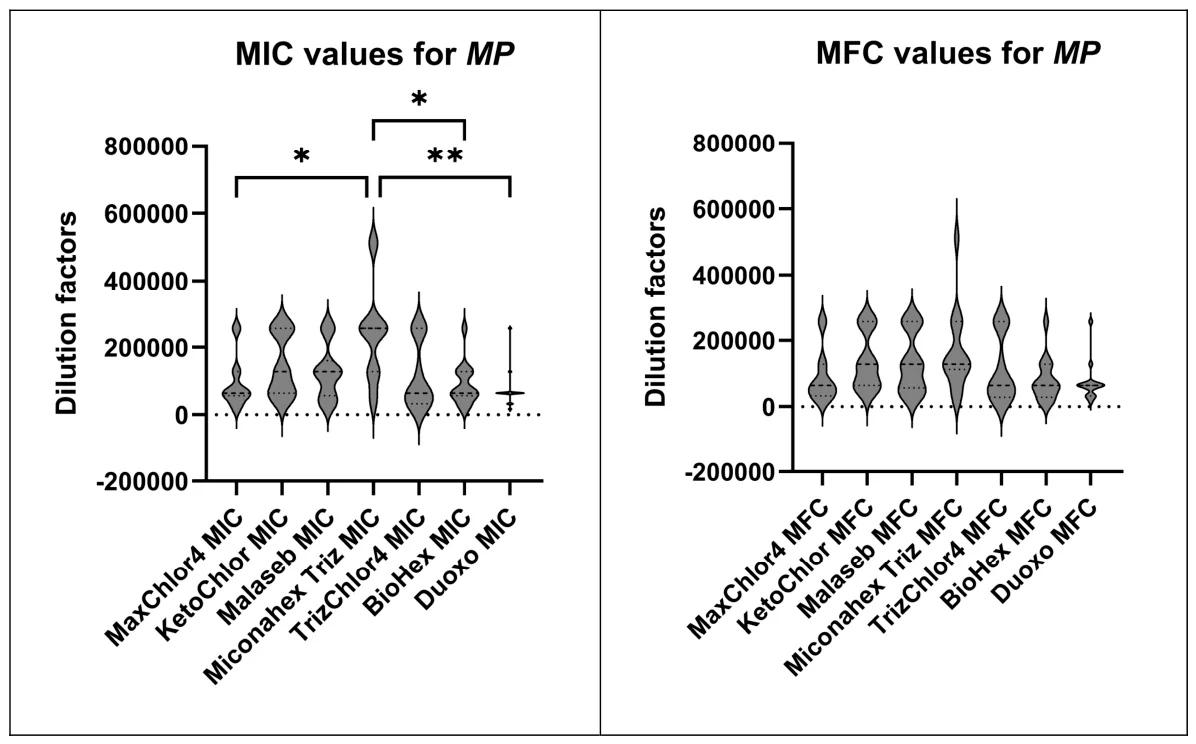
Violin plot representing comparisons of the minimum inhibitory concentration (MIC) and minimum fungicidal concentration (MFC) of MP isolates. * *p* < 0.05; ** *p* < 0.01. MP: *Malassezia pachydermatis*.

The *MP* ATCC strain (MA-14522) was inclued as an internal quality control. Malaseb^®^, KetoChlor^®^, MiconaHex+Triz^®^, and BioHex^®^ demonstrated MIC values of 1:128,000, whereas Douxo S3^TM^ Pyo and MAX Chlorexidine 4% demonstrated MIC values of 1:64,000. MFC values were 1:128,000 for Malaseb^®^ and KetoChlor^®^, and 1:64,000 for MiconaHex+Triz^®^, BioHex^®^, Douxo S3^TM^ Pyo, and MAX Chlorexidine 4%. TrizCHLOR^®^4 did not inhibit the ATCC strain at any of the tested solutions.

### 3.3. Comparison Between MDR-SP and MP

Inhibitory concentrations were largely similar between MDR-*SP* and *MP*. There was not a significant difference in MIC values for six of the seven shampoos tested (MAX Chlorhexidine 4%, Malaseb^®^, MiconaHex+Triz^®^, TrizCHLOR^®^4, BioHex^®^, and Douxo S3™ Pyo). KetoChlor^®^ was the only formulation that demonstrated a lower MIC for MDR-*SP* (1:32,000) when compared to *MP* (1:64,000) (*p* < 0.0001).

Comparing bactericidal and fungicidal activities, a significantly greater fungicidal activity was found for all the shampoos tested: MAX Chlorhexidine 4% (*p* < 0.0001), KetoChlor^®^ (*p* < 0.0001), Malaseb^®^ (*p* < 0.0001), MiconaHex+Triz^®^ (*p* = 0.0001), TrizCHLOR^®^4 (*p* = 0.013), BioHex^®^ (*p* < 0.0001), and Douxo S3™ Pyo (*p* < 0.0001).

## 4. Discussion

The results of this study showed that different chlorhexidine-based shampoos have very similar inhibitory, bactericidal, and/or fungicidal activity against MDR-*SP* clinical isolates and *MP* clinical isolates. In fact, although some statistical differences were detected using the present study design, such differences spanned, in most cases, only a single double dilution. Such difference is very likely to be clinically meaningless given the high dilution factors tested.

This study is also in agreement with previous studies [2,6] confirming that chlorhexidine-based shampoos exhibit similar inhibitory and bactericidal activity against *SP.* Furthermore, this study equally demonstrated that the selected shampoos demonstrate substantial in vitro antifungal activity against *MP* isolates. However, MIC and MFC endpoints were not reached within the tested dilution range for a limited number of *MP* isolates exposed to some formulations. To the authors’ knowledge, this is the first study to directly compare the antimicrobial activity of multiple chlorhexidine-based shampoos against both MDR-*SP* and *MP* isolates using standardized MBD methodology. While several previous studies have evaluated the efficacy of chlorhexidine-containing products against *SP* and MRSP isolates, information regarding the activity against *MP* and MDR-*SP* isolates remained limited.

This study also supports the recent guidelines for the treatment of canine pyoderma and *Malassezia* dermatitis suggesting the use of antimicrobial shampoos as a major treatment option for superficial skin infections. This, along with previous studies [1,7,11], supports the role of topical antiseptic therapy as part of antimicrobial stewardship strategies for the management of canine skin infections. Indeed, all shampoos performed well, reaching antimicrobial activity at very low concentrations. Such concentrations can be easily reached in clinical practice considering that chlorhexidine-based shampoos are recommended at doses ranging from 19 to 57 mL/m^2^ body surface area [11,12]. In fact, based on an average bathing volume of approximately 40 L per dog, chlorhexidine concentrations between 1:701 and 1:2105 are estimated to be reached during bathing [11]. However, it is important to keep in mind that the concentration of active ingredients is influenced during bathing, including several factors such as product dilution with water, rinsing volume, type of hair coat, contact time, presence of organic material, and shampoo formulation [7]. Conditions such as these are not tested by in vitro studies, like the one presented here. Similarly, the incubation periods used in this study were selected to allow standardized determination of MIC, MBC, and MFC endpoints under laboratory conditions, and were not intended to reproduce the contact time achieved during bathing [1,11].

Although Malaseb^®^ and MiconaHex+Triz^®^ shampoos had the lowest MIC and MBC/MFC values among the tested shampoos, they also demonstrated the greatest variability between MIC and MBC/MFC. However, because MIC determinations are based on serial two-fold dilutions, such variability is unlikely to be clinically relevant given the high dilution ratio tested. In addition, though statistically significant, the differences between shampoos were limited to only a single two-fold dilution and for this reason are probably clinically irrelevant. Outside of the minor differences observed, the bactericidal and antifungal activity of all the shampoos tested had similar results for MBC_50_/MFC_50_ and, more importantly, for MBC_90_/MFC_90_. Nevertheless, it is noteworthy that the MIC and MFC against *MP* were not achieved for some shampoos, limiting the calculation of MIC_90_ and MFC_90_. These findings may also reflect individual variability in susceptibility or a true resistance to azoles and/or chlorhexidine. Previous studies have reported reduced susceptibility, tolerance, and resistance-associated mechanisms in some *Malassezia* isolates, especially if previously exposed to antifungal agents [5]. These findings can explain the anecdotal lack of clinical efficacy of topicals in the treatment of *Malassezia* dermatitis. Additional studies are needed to determine mechanisms that reduce susceptibility to antimicrobial agents [2,5].

The results of this study also demonstrated that all shampoos exhibit antibacterial and antifungal activity against MDR-*SP* and *MP* at clinically achievable concentrations regardless of the chlorhexidine concentration or the presence or absence of an antifungal agent. In fact, the chlorhexidine concentrations ranged from 2% to 4%, and most formulations contained chlorhexidine gluconate, with only one of them utilizing chlorhexidine digluconate (Douxo S3™ Pyo). Altogether, these data confirm the findings of previous studies [1,7,10,13] suggesting that more than the chlorhexidine concentration or the additional presence of azoles and tris-EDTA, differences among products may more likely be related to the different formulations of shampoos, including inactive ingredients such as excipients, surfactants, and vehicle composition.

As mentioned above, the group of shampoos tested here demonstrated high levels of inhibitory activity against MDR-*SP* at dilutions from 1:32,000 to 1:128,000, with concentrations of chlorhexidine ranging from 0.156 µg/mL to 0.937 µg/mL. Dilutions from 1:16,000 to 1:32,000, equivalent to chlorhexidine concentrations ranging from 0.625 µg/mL and 2.5 µg/mL, were needed to kill MDR-*SP*. All shampoos also demonstrated very high inhibitory activity against *MP* at dilutions from 1:16,000 to 1:128,000, with concentrations of chlorhexidine ranging from 0.156 µg/mL to 2.5 µg/mL. The antifungal activity (MFC) of all shampoos was also very high, with dilutions ranging from 1:64,000 to 1:128,000, with concentrations of chlorhexidine ranging from 0.156 µg/mL to 2.5 µg/mL. Interestingly, when the antifungal action (MFC) of each shampoo was compared with their own antibacterial (MBC) activity, a significantly higher antimicrobial action was seen against *MP* compared to MDR-*SP*. These data are somewhat in line with the results reported by Bergen et al. (2024) [11], demonstrating an antimicrobial effect of a 4% chlorhexidine-based shampoo against MDR-*SP* and *MP* at dilutions of 1:128,000 (equivalent to 0.3125 µg/mL) (MIC) and 1:64,000 (equivalent to 0.625 µg/mL) (MBC) for MDR-*SP* and 1:80,000 (equivalent to 0.5 µg/mL) (MIC) and 1:40,000 (equivalent to 1 µg/mL) (MFC) for *MP* isolates. The inhibitory activity of all the shampoos was comparable between MDR-*SP* and *MP* with no significant differences found, except for KetoChlor^®^ (*p* < 0.0001). The reason for this difference in vitro of KetoChlor^®^ compared with other shampoos is unknown and somewhat surprising, especially considering that a significant synergy has been observed between chlorhexidine and ketoconazole when compared with chlorhexidine and miconazole or chlorhexidine alone against MDR-*SP* and *MP* [1]. The combination of chlorhexidine with azoles has demonstrated inhibitory activity against *Malassezia* organisms [13,14]. Formulations of azoles with chlorhexidine helps to extend the coverage of a product to aid in the treatment of azole-resistant infections, as most *MPs* are susceptible to chlorhexidine [13,15].

A limitation of the present study is that antimicrobial activity was evaluated only under planktonic conditions. Although broth microdilution is a reference method for susceptibility testing, both *SP* and *MP* have been shown to form biofilms, particularly chronic and recurrent infections. The presence of biofilm protects microorganisms, making them less susceptible to antimicrobial agents, and for this reason, the activity observed in this study may not fully reflect the response of biofilm-associated infections to topical therapy. Because biofilm formation may influence the susceptibility profile, future studies should investigate the activity of such shampoos on microorganisms in biofilm state: specifically, assessing the efficacy of such products in disrupting and/or inhibiting the formation of biofilms compared with the planktonic state.

## 5. Conclusions

This study demonstrated comparable antimicrobial activity across the selected shampoos regarding either their bactericidal or fungicidal activity at the concentrations tested. However, MIC and MFC endpoints were not reached for a limited number of *MP* isolates exposed to some formulations, indicating variability in susceptibility among clinical isolates. It is also important to consider that independently from the product, when the concentration of chlorhexidine needed to inhibit or kill MDR-*SP* and *MP* was analyzed for each shampoo, very similar values were seen. In particular, the two 4% chlorhexidine shampoos and the 3% chlorhexidine shampoo performed similarly or slightly inferiorly than the 2% chlorhexidine products against both MDR-*SP* and *MP*.

The antimicrobial activity of all tested shampoos was maintained across a broad range of dilution conditions relevant to routine clinical shampoo application. Because this is an in vitro study, factors such as contact time, skin microenvironment, biofilm formation, presence of organic debris, and patient compliance were not evaluated. Nevertheless, the results demonstrated that the concentrations of antimicrobial agents in the tested shampoos can be clinically achievable. Further clinical studies are needed to investigate the in vivo effects of the shampoos in therapeutic efficacy.

## Data Availability

The original contributions presented in this study are included in the article/Supplementary Material. Further inquiries can be directed to the corresponding author.

## Supplementary Materials

The following supporting information can be downloaded at: https://www.mdpi.com/article/10.3390/microorganisms14081660/s1, Table S1: Susceptibility of multidrug-resistant (MDR) *Staphylococcus pseudintermedius* clinical isolates, *n* = 30. PBP2a: Penicillin-binding protein; MIC: minimum inhibitory concentration; CLSI: Clinical and Laboratory Standards Institute [3,9].
