# Supplementary material for: In Vitro Evaluation of the Antimicrobial Activity of Chlorhexidine-Based Shampoos Against Clinical Isolates of Malassezia pachydermatis and Multidrug-Resistant Staphylococcus pseudintermedius
Source: Microorganisms. 2026 Jul 29;14(8):1660. doi: 10.3390/microorganisms14081660 (PMC13515812; doi:10.3390/microorganisms14081660)
Supplement: Supplementary file 1 [file microorganisms-14-01660-s001.zip › microorganisms-4437718-supplementary.pdf]

	S. pseudintermedius	
Antimicrobial	MIC µg/mL	CLSI Interpretation
Amikacin	≤4	S 1/30 R 29/30
Amoxicillin/Clavulanic Acid	0.5	S 1/30 R 29/30
Ampicillin	>1	R 30/30
Cefazolin	<1	S 1/30 R 29/30
Cefovecin	2	R 30/30
Cefpodoxime	≤2	R 30 /30
Cephalothin	≤2	R 30/30
Chloramphenicol	≤ 2	S17/30 R 13/30
Clindamycin	>4	S 3/30 R 27/30
Doxycycline	<0.12	R 30/30
Enrofloxacin	>2	S 4/30 R 26/30
Erythromycin	>4	S 3/30 R 27/30
Gentamicin	>8	S12/30 R 18/30
Imipenem	<1	S 2/30 R 28/30
Marbofloxacin	>2	S 7/30 R 23/30
Minocycline	<0.5	R 30/30
Oxacillin + 2% NaCl	≤0.25	S 1/30 R 29/30
Penicillin	>8	S 1/30 R 29/30
Rifampin	≤1	S 24/30 R 6/30
Tetracycline	>1	S 1/30 R 29/30
Trimethoprim/Sulfamethoxazole	>4	S 8/30 R 22/30
PBP2a agglutination	Positive 30/30	Negative 0/30
mecA gene	Positive 29/30	Negative 1/30

Table S1. Susceptibility of multidrug resistant (MDR)[1] *Staphylococcus pseudintermedius* clinical isolates, n= 30. PBP2a: Penicillin binding protein; MIC: Minimum inhibitory concentration; CLSI: Clinical and Laboratory Standards Institute[2].

1. Magiorakos A, Srinivasan A, Carey R et al. Multidrug-resistant, extensively drug resistant and pandrug-resistant bacteria: an international expert proposal for interim standard definitions for acquired resistance. *Clin Microbiol Infect* 2012; **18**: 268–281.
2. CLSI. Performance Standards for Antimicrobial Disk and Dilution Susceptibility Tests for Bacteria Isolated From Animals. 7th ed. CLSI supplement VET01S. Clinical and Laboratory Standards Institute; 2024
